# Quantitative Proteomic Analysis of Plasma Exosomes to Identify the Candidate Biomarker of Imatinib Resistance in Chronic Myeloid Leukemia Patients

**DOI:** 10.3389/fonc.2021.779567

**Published:** 2021-12-21

**Authors:** Mei-Yong Li, Cui Zhao, Lian Chen, Fang-Yi Yao, Fang-Min Zhong, Ying Chen, Shuai Xu, Jun-Yao Jiang, Yu-Lin Yang, Qing-Hua Min, Jin Lin, Hai-Bin Zhang, Jing Liu, Xiao-Zhong Wang, Bo Huang

**Affiliations:** ^1^ Jiangxi Province Key Laboratory of Laboratory Medicine, Department of Clinical Laboratory, The Second Affiliated Hospital of Nanchang University, Nanchang, China; ^2^ Huanggang Central Hospital Affiliated to Changjiang University, Huanggang, China; ^3^ Department of Ultrasound, The First Affiliated Hospital of Nanchang University, Nanchang, China

**Keywords:** exosomes, chronic myeloid leukemia, proteomics, imatinib, ribosomal protein L13, ribosomal protein L14

## Abstract

**Background:**

Imatinib (IM), a tyrosine kinase inhibitor (TKI), has markedly improved the survival and life quality of chronic myeloid leukemia (CML) patients. However, the lack of specific biomarkers for IM resistance remains a serious clinical challenge. Recently, growing evidence has suggested that exosome-harbored proteins were involved in tumor drug resistance and could be novel biomarkers for the diagnosis and drug sensitivity prediction of cancer. Therefore, we aimed to investigate the proteomic profile of plasma exosomes derived from CML patients to identify ideal biomarkers for IM resistance.

**Methods:**

We extracted exosomes from pooled plasma samples of 9 imatinib-resistant CML patients and 9 imatinib-sensitive CML patients by ultracentrifugation. Then, we identified the expression levels of exosomal proteins by liquid chromatography-tandem mass spectrometry (LC-MS/MS) based label free quantification. Bioinformatics analyses were used to analyze the proteomic data. Finally, the western blot (WB) and parallel reaction monitoring (PRM) analyses were applied to validate the candidate proteins.

**Results:**

A total of 2812 proteins were identified in plasma exosomes from imatinib-resistant and imatinib-sensitive CML patients, including 279 differentially expressed proteins (DEPs) with restricted criteria (fold change≥1.5 or ≤0.667, p<0.05). Compared with imatinib-sensitive CML patients, 151 proteins were up-regulated and 128 proteins were down-regulated. Bioinformatics analyses revealed that the main function of the upregulated proteins was regulation of protein synthesis, while the downregulated proteins were mainly involved in lipid metabolism. The top 20 hub genes were obtained using STRING and Cytoscape, most of which were components of ribosomes. Moreover, we found that RPL13 and RPL14 exhibited exceptional upregulation in imatinib-resistant CML patients, which were further confirmed by PRM and WB.

**Conclusion:**

Proteomic analysis of plasma exosomes provides new ideas and important information for the study of IM resistance in CML. Especially the exosomal proteins (RPL13 and RPL14), which may have great potential as biomarkers of IM resistance.

## Introduction

Chronic myeloid leukemia (CML) is a malignant myeloproliferative disease characterized by Philadelphia (Ph) chromosome produced by reciprocal translocation between chromosomes 9 and 22 [t (9,22)] (1). The Ph chromosome can be transcribed and encoded into BCR-ABL1 oncoprotein, a constitutive tyrosine kinase that promotes abnormal cellular proliferation and disease progression by stimulating multiple downstream signaling pathways (2). Imatinib (IM), a tyrosine kinase inhibitor (TKI) that target the ATP-binding site of BCR-ABL kinase, has become first-line treatment and effectively improved the survival for the majority of CML patients (3). However, 20-30% of CML patients will develop resistance to IM (4). Hence, there is a desperate need to discover new diagnosis biomarkers of CML and potential targets for CML therapeutics.

Exosomes are nanometer-sized extracellular vesicles (EVs) which are approximately 40-160 nm in diameter (average ~100 nm) (5). When endosomes or multivesicular bodies (MVBs) fuse with the plasma membrane, the exosomes are released (6). The role of exosomes was initially thought to be the carrier of unnecessary cellular waste that remove excess constituents from cells to maintain cellular homeostasis (7). Currently, increasing studies have reviewed that exosomes can carry many functional molecules including proteins, lipids, and nuclear acids (DNA, mRNA, lncRNA, miRNA, and circRNA) derived from the parent cell (8), which serve an essential role in regulating intercellular communication during different physiological and pathological processes (9). In addition, various substances enclosed within exosomes are wrapped by the lipid bilayer, which is relatively stable in body fluids (blood, ascites, saliva, urine, and so on) (10). Due to these unique features of exosomes, they have become ideal candidate biomarkers for the diagnosis, prognosis, and response to therapy of many malignant tumors.

A growing body of research has shown that the exosome is an important part of the tumor microenvironment implicated in chemoresistance development in cancer (11, 12). As for studies related to IM resistance in CML, our previous study found that exosomal miR-365 derived from imatinib-resistant (IM-R) cells can be directly transferred to imatinib-sensitive (IM-S) cells and to confer drug-resistance traits (13). Hrdinova and coworkers reported a detailed characterization of the protein cargo of the exosomes derive from K562 and K562IR cells and found IFITM3, CD146 and CD36 are specific exosomal markers associated with the IM resistance (14). However, the single-cell model of CML may not fully reflect the complexity and heterogeneity of CML *in vivo*. Therefore, we conceive that it is necessary to identify plasma exosomal proteins for finding of reliable biomarkers and potential therapeutic targets associated with the IM resistance in CML.

In this study, we acquired a detailed proteomic profile of exosomes derived from plasma of IM-R and IM-S CML patients. Differentially expressed proteins (DEPs) identified were associated with ribosome, acute myeloid leukemia, prostate cancer, and cholesterol metabolism. We subsequently screened multiple potential biomarkers and verified several in independent cohorts. Our study might be useful for further studies of novel diagnostics and therapeutics for chemoresistance of IM in CML patient.

## Materials And Methods

### Patients and Plasma Samples

All patients were recruited from the Second Affiliated Hospital of Nanchang University (Nanchang, China). The diagnoses, treatment responses, and relapse criteria of CML were made according to the criteria of World Health Organization (15) and the guidelines for diagnosis and treatment of chronic myelogenous leukemia in China (2016 edition) (16). The subjects enrolled in the current study were from newly diagnosed CML patients and none of the patients had received prior TKI therapy or other medical treatment of CML before plasma samples were obtained. Pooled plasma samples from 9 IM-R and 9 IM-S CML patients were used for discovery proteomics analysis. Then, plasma samples from 18 IM-R and 18 IM-S CML patients were used to validate candidate markers. Detailed information of all participants is given in **Supplementary Table S1**. Peripheral venous blood sample from every single participant was collected in the fasting state and plasma was separated by centrifugation at 2,000 × g for 15 min. The plasma supernatant without lipidemia or hemolysis was collected and stored at -80˚C for exosome isolation. This study was conducted in accordance with the Declaration of Helsinki and was approved by the Medical Ethics Committee of the Second Affiliated Hospital of Nanchang University. All patients provided written informed consent.

### Exosome Isolation

We isolated exosomes from plasma samples through ultracentrifugation as in our earlier report with slight modifications (17). The experimental workflow is shown in **Figure 2A**. The plasma samples were diluted twice with PBS to reduce the viscosity. The mixed liquid was centrifuged to remove cells and cell debris by 10 min centrifugation at 400 × g (4°C), followed by 20 min centrifugation at 2000× g (4°C). Subsequently, the resultant supernatant was collected and centrifuged at 10,000 × g for 30 min (4°C) and then was filtered through a 0.22 µm syringe membrane filter (Millipore, USA). The supernatant was transferred into ultracentrifuge (UC) tubes with a total volume of 10 ml and ultracentrifuged at 110,000 × g (4°C) for 70 min (OptimaL-80XP, SW41 rotor, Beckman Coulter, USA) to enrich exosomes. The pellet was resuspended with cold PBS and centrifuged again at 110,000 × g for 70 min (4°C). The final exosomes was resuspended in 50 µl PBS for subsequent analysis.

### Nanoparticle Tracking Analysis (NTA)

The exosomes were appropriately diluted with 1×PBS buffer, then the size distribution and concentration of exosome were measured by NTA by using a ZetaView PMX 110 instrument (Particle Metrix, Meerbusch, Germany). The exosome solutions were illuminated by a 488nm laser, and the movement of nanoparticles caused by Brownian motion was recorded for 60 seconds and the average frame rate was 20 frames per second. NTA measurement was recorded and analyzed at 11 positions with the corresponding software (ZetaView 8.04.02 SP2).

### Transmission Electron Microscope (TEM)

The transmission electron microscope was used to visualize(observe) the morphology of exosome with negative staining. Briefly, the extracted exosomes were fixed in 2% paraformaldehyde. Then 10 μl of the exosome sample was dropped onto a formvar-coated 200-mesh copper grids for 1 min at room temperature, and the excess solution was blotted with filter paper. The grid of absorbed exosomes was treated with 2% uranyl acetate and remaining uranyl acetate was wicked away with filter paper after 1 min. Finally, the dried grid was examined by a TEM (Philips CM100, FEI, Eindhoven).

### Western Blot Analysis (WB)

Total proteins of exosomes were extracted by RIPA buffer (P0013B, Beyotime, China) with 1:100 volume of PMSF. The exosome-specific protein, RPL13, and RPL14 were verified by WB. Exosomal proteins were electrophoresed by 10% sodium dodecyl sulfate-polyacrylamide gel electrophoresis (SDS-PAGE). Separated proteins were transferred to a nitrocellulose membrane (0.2μm, Millipore, Billerica, USA) and blocked for 60 minutes at room temperature with 5% non-fat dry milk in Tris-buffered saline containing 0.2% Tween-20 (TBST). After blocking, the membrane was incubated with primary antibody overnight at 4°C, including anti-Alix (Santa Cruz Biotechnology, sc-271975, 1:500), anti-TSG101 (Proteintech, 28283-1-AP, 1:6000), anti-CD81 (Proteintech, 66866-1-Ig, 1:3000), anti-RPL13 (Abcam, ab134961, 1:6000), anti-RPL14 (Abcam, ab181200, 1:6000). Then, the membranes were washed thrice (10 minutes each time) with TBST for shaking for each time and incubated with horseradish peroxidase-conjugated secondary antibodies (Boster Biological Technology, 1:6000) for 1 h at room temperature. The immunoreactive blots were visualized with Pro-light HRP chemiluminescence kit (Tiangen, China) using a ChemiDoc XRS System (Bio-Rad).

### Exosomal Protein Extraction and Digestion

Exosomes were solubilized in lysis buffer (8 M urea and 1% protease inhibitor) and sonicated three times at high intensity using an ultrasonic processor (Scientz). The suspension was centrifuged at 12,000 × g (4°C, 15 min) to remove debris. Then, the supernatant was gathered and measured with BCA protein quantification kit. To digest proteins, proteins were first reduced by 5 mM dithiothreitol (56°C, 30 min), followed by alkylated with 11 mM iodoacetamide (room temperature, 15 min in darkness). Furthermore, the sample was diluted to a final urea concentration to < 2M by adding 100 mM triethylammonium bicarbonate buffer (TEAB). Finally, proteins were digested twice with trypsin, the first digestion were performed with trypsin (overnight, 37°C) at a ratio of 1:50 (enzyme:protein) and 1:100 for the second digestion (4h).

### Liquid Chromatography-MS/MS Analysis

Digested samples were subjected to LC-MS/MS detection using a minor modifications of previously described method by Hou et al (18). The tryptic peptide mixture was resuspended in solvent A (0.1% formic acid) and directly inject into a home-made reversed-phase analytical column (15 cm length, 75 μm i.d.). Then, the peptides were separated on an EASY-nLC 1000 UPLC system (Thermo Fisher Scientific) with a linear gradient mobile phase buffer at constant flow rate of 400 nL min^-1^. The chromatography gradient was carried out starting at 6% solvent B (0.1% formic acid in 98% acetonitrile), increased to 23% B over 26 min, changed to 35% in 8 min, climbing to 80% in 3 min and maintenance at 80% for an additional 3 min. The eluted peptides were loaded into the nanospray ionization (NSI) source and performed tandem mass spectrometry (MS/MS) analysis on a Q Exactive™ Plus (Thermo Fisher Scientific) coupled online to the UPL (Thermo Fisher Scientific). The applied voltage of electrospray was 2.0 kV. The intact peptides were detected for MS full scans with the scanning range of m/z was 350 to1800 and a resolution of 70,000 in the Orbitrap. Subsequently, the selected peptides were detected at a resolution of 17,500 and normalized collision energy (NCE) of 28. The MS analysis was performed by a data-dependent scan mode that alternated between one MS scan and 20 MS/MS scans, and the dynamic exclusion was set to 15.0 s. Automatic gain control (AGC) target value and fixed first mass were set to 5E4 and 100 m/z, respectively.

### Database Search

The secondary mass spectrum data were retrieved using Proteome Discoverer (PD) 2.4 software (Thermo Scientific) with the Maxquant search engine (v.1.5.2.8). In detail, the raw MS/MS data files were searched against the human UniProt/SwissProt proteome database with reverse decoy database. The cleavage enzyme was set as Trypsin/P, and the number of missed cleavages was allowed up to two. The length of minimal peptide specified to be 7 amino acid residues, the maximal modification number of allowed per peptides was set as 5. Precursor mass tolerance were set to 10 ppm and 5 ppm for the first search and the main search, respectively. The fragment ion mass tolerance was as 0.02 Da. The acetylation modification on N-terminal and oxidation modification on methionine were designated as variable modifications. The carbamidomethyl modification on cysteine residues was designated as a fixed modification. The quantitative method of proteins was label-free quantification (LFQ), and the false discovery rate (FDR) of protein identification and peptide-spectrum match (PSM) identification was set as 1%.

### Bioinformatics Analyses

Gene Ontology (GO) annotation proteome was obtained based on the UniProt-GOA database (http://www.ebi.ac.uk/GOA/). The fold change values ≥ 1.5 for the upregulated proteins and ≤ 0.667 for the downregulated, which were used as the criteria to identify the DEPs between IM-R and IM-S samples. We performed the bioinformatics analyses in order to further analyze the biological function of DEPs. For GO functional enrichment analysis, proteins were categorized into three ontologies (biological process, molecular function and cellular compartment) using the extension R package ‘ClusterProfiler’ (19)(https://www.bioconductor.org/packages/release/bioc/html/clusterProfiler.html). We used the Kyoto Encyclopedia of Genes and Genomes (KEGG) database (http://www.genome.jp/kegg/) to perform pathway enrichment analysis. The enrichment of DEPs was detected by double-tailed Fisher’s exact test. The adjusted p-value <0.05 for enrichment analysis was considered significant. Protein interaction networks were analyzed by STRING (20) (https://string-db.org/,v.11.0), and the parameter settings were as follows: the minimum required interaction scores were set at highest confidence (0.900), the network display options were set at hide disconnected nodes in the network, the other parameters were set to default values. The essential hub proteins screens were performed by Cytoscape software (21).

### PRM-MS Analysis

Twenty proteins were screened through bioinformatics analyses, and further verified with PRM. The digested peptides were dissolved in 0.1% formic acid (mobile phase A) and analyzed using PRM method on Exploris 480™ mass spectrometer (Thermo) coupled to an Easy-NLC 1200 UPLC-system (ThermoFischer). The detection and analysis of peptide parent ions and their secondary fragments were performed in the Orbitrap analyzers. Data was acquired with Targeted Mass modes. Higher-energy collision dissociation (HCD) was set at normalized collision energy of 27%. Mass spectrometer settings were, for full MS acquisitions, mass-to-charge ratio (m/z) range set at 395-815 m/z, resolution set at 60,000, automatic gain control (AGC) set at 300%, maximum ion injection time of 100 ms; for MS/MS acquisitions, resolution set at 15,000, automatic gain control (AGC) set at 75%, maximum ion injection time set to 100 ms, isolation window set to 1.6 m/z.

### Statistical Analyses

All other data were analyzed using GraphPad Prism (version 8.0.2; La Jolla, CA, USA). The Mann-Whitney U-test was used to compare differences of the exosomal RPL13 and RPL14 levels between IM-R and IM-S CML patients. The values of p < 0.05 (two-sided) were considered statistically significant.

## Results

### General Experiment Design

**Figure 1:**
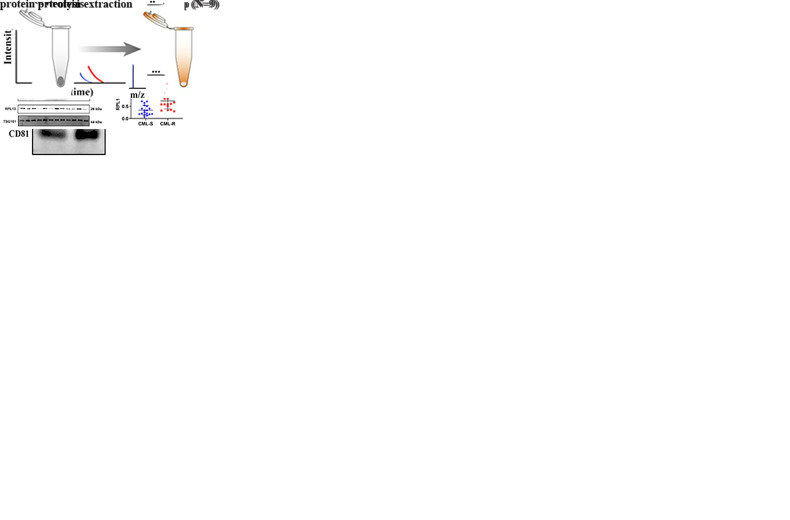
Schematic workflow shown the LC-MS/MS-based quantitative proteomic analysis of exosome isolated from plasma samples of IM-R and IM-S CML patients. CML, chronic myeloid leukemia; CP, chronic phase; BP, blastic phase; AP, acceleration phase; TEM, transmission electron microscope; NTA, nanoparticle tracking analysis; WB, western blot; LC-MS/MS, liquid chromatography-tandem mass spectrometry.

We designed a simple workflow to understand the protein composition of the CML plasma exosomes and to screen potential diagnostic exosomal protein biomarkers for IM-R CML patients, as illustrated in **Figure 1**. For proteomics analysis, plasma samples obtained from 9 IM-R and 9 IM-S CML patients were pooled, respectively. Exosomes were isolated from the pooled plasma samples using ultracentrifugation method and verified using TEM, NTA, and WB. The gained exosomes were lysed to extract proteins and were digested into peptides by trypsin. The exosomal protein profiles were analyzed by label free quantitative proteomics based on LC-MS/MS. DEPs and candidate protein biomarkers were determined by bioinformatics analyses, and then PRM analyses were applied to validate the candidate proteins. Based on the validation results, two proteins were selected as diagnostic markers and the expression levels of the selected proteins were further validated by WB in 18 IM-R and 18 IM-S CML patients.

### Characterization of Plasma Exosomes

**Figure 2:**
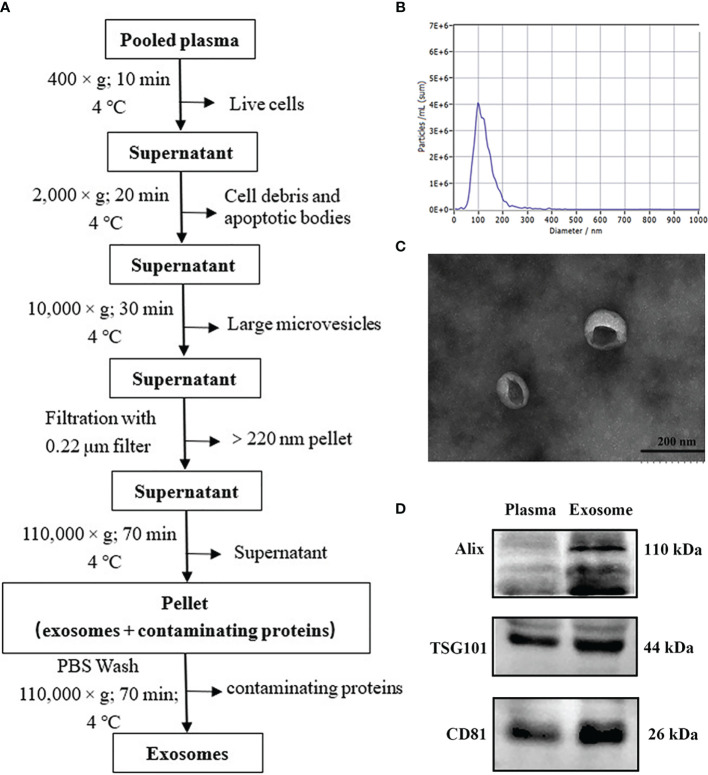
Isolation and validation of human plasma exosomes. **(A)** Exosome purification flow chart from plasma by ultracentrifugation. **(B)** Transmission electron micrograph of exosomes from plasma (bar size: 200 nm). **(C)** The concentration and size distribution of plasma exosomes analyzed by nanoparticle tracking analysis (NTA). **(D)** Purified plasma exosomes were detected using western blot for exosome-specific markers (ALIX, TSG101, and CD81).

**Figures 2B–D** show representative images of plasma exosomes obtained with TEM, NTA, and WB. NTA results revealed the average size of the exosomes was approximately 100 nm in diameter (**Figure 2B**). TEM demonstrated that the exosomes took on cup-shaped membrane vesicles and the sizes consistent with the NTA results (**Figure 2C**). We further confirmed the presence of characteristic exosomal membrane markers including ALIX, TSG101, and CD81 by WB. Altogether, these results illustrated that exosomes from clinical plasma samples were successfully collected and purified.

### Proteomic Profiling of Exosomes From IM-R and IM- S CML Patients

**Figure 3:**
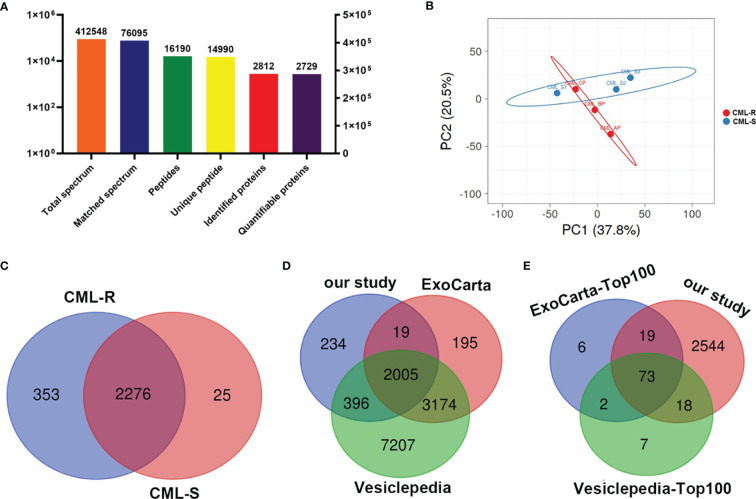
Protein identification and quantitative evaluation of plasma derived exosomes. **(A)** The results distribution diagram of LC-MS/MS. **(B)** Principal component analysis plot shown differences in the proteome profiles between CML-R and CML-S group. **(C)** The Venn diagram displays the number of exosomal proteins identified in CML-R and CML-S patients. **(D, E)** The Venn diagram displays the comparison between proteins identified in the present study and those identified in the exosomal database (Exocarta and Vesiclepedia).

In order to achieve the characteristics of exosome proteome in the IM-R and IM-S CML patients, protein profiling was performed on the extracted plasma exosomes. 14990 unique peptides were identified from plasma exosomes in all groups, representing 2812 proteins, including 2729 quantifiable proteins (**Figure 3A** and **Supplementary Table 2**). Principal component analysis of the data revealed a group of data (CML-S1) in the sensitive group with a large degree of dispersion (**Figure 3B**), and this group was removed during further analysis. As shown in **Figure 3C** and **Supplementary Table 3**, a total of 2654 quantifiable proteins were identified in all groups. Among them, 2276 proteins (85.8%) overlapped between the IM-R and IM-S group, 353 (13.3%) and 25 (0.9%) proteins were found in IM-R and IM-S group, respectively. In order to verify the relationship between the plasma exosomal proteins and known vesicular proteins, we compared quantified exosomal proteins from the two plasma groups with the proteins in Exocarta (22, 23) and Vesiclepedia databases (24, 25). The result was displayed as Venn diagram in **Figure 3D**. Of the 2654 identified plasma exsomal proteins, 2005 (75.55%) proteins were shared by the three data sets, and 234 (8.82%) proteins are only present in our study, which may be newly discovered exosomal proteins. In addition, out of the top 100 reported extracellular vesicle markers in ExoCarta, and Vesiclepedia database, 73 proteins were identified in our study (**Figure 3E**). These data confirmed that the exosomes we prepared are rich in exosomal proteins.

### GO Classification and KEGG Pathway Analysis of DEPS

**Figure 4:**
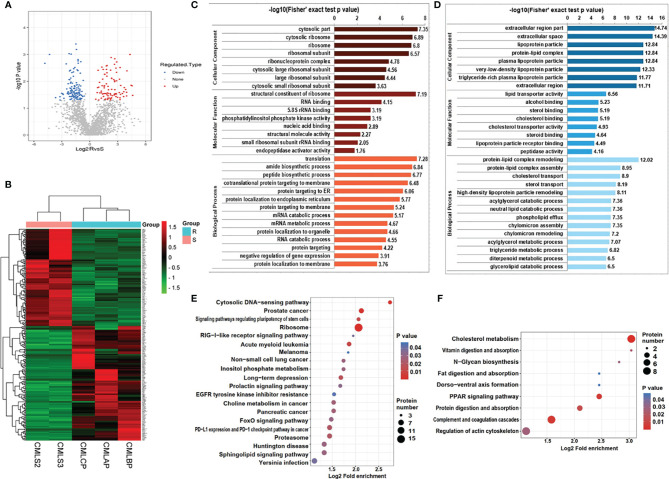
Bioinformatics analysis of DEPs in plasma exosomes derived from IM-R and IM-S CML patients. **(A)** The volcano plots showing significantly differential expressed proteins include 151 upregulated proteins (colored in red) and 128 downregulated proteins (colored in blue) based on fold changes (≥1.5 or ≤0.667 and p<0.05). **(B)** The heatmap plots of DEPs. **(C, D)** GO enrichment analysis of DEPs. Red bar and blue bar represent enriched GO terms of up-regulated proteins **(C)** and down-regulated proteins **(D)** respectively. **(E, F)** The result of KEGG pathway enrichment analyses of DEPs was presented in a bubble plot, including upregulated proteins **(E)** and downregulated proteins **(F)**. Each colored bubble indicated different pathways.

In order to explore the difference of exosomal proteins between the IM-R group and the IM-S group, a fold change ≥1.5 or ≤ 0.667 and p value <0.05 was used as a threshold to identify DEPs. As illustrated in the volcano plot and heat map (**Figures 4A, B**, and **Supplementary Table 4**), we screened 279 DEPs under this condition, including 151 upregulated proteins and 128 downregulated proteins. To analyze the biological function of the DEPs further, we conducted GO Term and KEGG pathway enrichment analysis. GO analysis results were shown in **Figures 4C** **, D**. Cellular component (CC) analysis revealed that the upregulated proteins were enriched in cytosolic part, cytosolic ribosome, ribosome, and ribsomal subunit, whereas the downregulated proteins were enriched in extracellular region part, extracellular space, and lipoprotein particle. As for molecular functions (MF), the upregulated proteins were associated with structural constituent of ribosome, whereas the downregulated proteins were associated with lipid transporter activity. As for biological processes (BP), the upregulated proteins were involved in translation, amide biosynthetic process, and peptide biosynthetic process, whereas the downregulated proteins were involved in protein-lipid complex remodeling, protein-lipid complex assembly, and cholesterol transport. Through KEGG pathway annotations, we found that ribosome, acute myeloid leukemia and prostate cancer were the most enriched pathways involved in the upregulated proteins (**Figure 4E**), while the downregulated proteins were enriched in pathways associated with cholesterol metabolism (**Figure 4F**). The functional analysis showed that the upregulated and downregulated proteins were related to ribosome and lipid metabolism, respectively. More detailed results of GO Term and KEGG pathway enrichment analysis can be obtained from **Supplementary Table 5**, **6**.

### Interaction Network Construction of DEPs and Identification of Key Proteins

**Figure 5:**
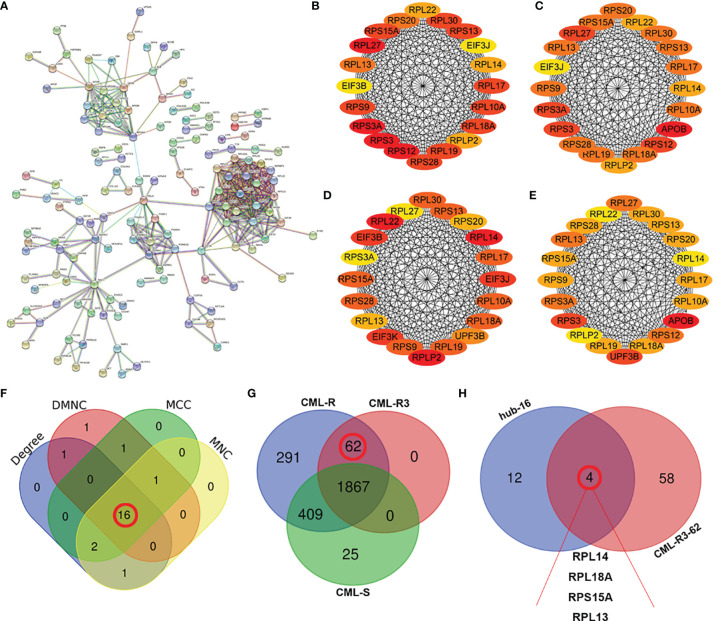
Screening of hub proteins for imatinib resistance **(A)** Protein–protein interaction (PPI) networks of DEPs. **(B–E)** The top 20 proteins in cytoHubba plugins: Stress **(B)**, Closeness **(C)**, EcCentricity **(D)**, and MCC **(E)**. **(F)** Venn diagram of the top 20 genes by different algorithms. The numbers (16) in the red circles indicate the common hub proteins identified by four algorithms. **(G)** Comparison of the numbers of proteins in CML-R, CML-R3 and CML-S group. The numbers (62) in the red circles indicate 62 proteins that only exist in IM-R group (CML-AP, CML-BP, and CML-CP). **(H)** Venn diagrams of 16 hub proteins and 62 CML-R3 proteins. The four proteins (RPL13, RPL18A, RPS15A, and RPL13 were screened as candidate proteins. DEPs, differentially expressed proteins; CML-R, Proteins identified in the imatinib resistant group; CML-S, Proteins identified in the imatinib sensitive group; CML-R3, Proteins identified in the all imatinib resistant group.

To investigate the interactions of DEPs, we constructed a comprehensive network using the STRING database with high confidence expressions (scores >0.9) and visualized with Cytoscape 3.8.1. In total, there are 155 DEPs in the PPI network, which contains 83 upregulated and 72 downregulated proteins (**Figure 5A**). Next four different algorithms (MCC, MNC, DMNC, and Degree) of the cytoHubba plugin were used to screen the hub proteins (**Figures 5B–E**) and 16 proteins are the common hub proteins (**Figure 5F**). **Figure 5G** is a venn diagram consisting of three groups, 62 proteins in the red box are present in every pooled IM-resistant CML samples and are not present in IM-sensitive CML samples. Finally, 16 common hub proteins overlapped with 62 proteins expressed only in the RR group and 4 proteins (RPL14, RPL18A, RPS15A, and RPL13) were screened to as candidate proteins, as shown in **Figure 5H**.

### Verification of Candidate Biomarkers with PRM and Western Blot Analysis

**Figure 6:**
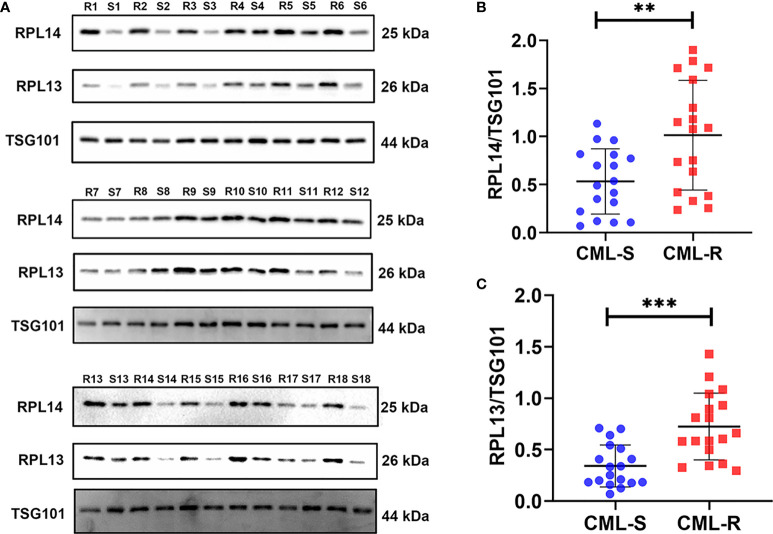
Validation of plasma exosomal proteins RPL13 and RPL14 in independent cohorts. **(A)** The expression levels of RPL13 and RPL14 were detected by western blot in plasma exosome samples from 18 CML-R and 18 CML-S patients. TSG101 serves as an internal control for equivalent amounts of protein (15 μg). **(B, C)** The scatter diagram shown the quantified data of RPL14 **(B)** and RPL13 **(C)**, respectively. CML-S, imatinib-sensitive chronic myeloid leukemia patients; CML-R, imatinib-resistant chronic myeloid leukemia patients. **p < 0.01, ***p < 0.001.

To validate the candidate proteins and the MS results, we selected twenty proteins including four candidate proteins for quantification using PRM. Of the 20 proteins, 13 proteins were detected and the fold difference was consistent with label-free quantification. In particular, the candidate proteins RPL14, RPS15A, and RPL13 were only identified in IM-R CML patients, and not in IM-S CML patients. Based on the fold change as well as the result of PRM, candidate exosomal proteins RPL14, and RPL13 were selected for validation by WB analysis in individual samples ( 18 IM-R and 18 IM-S CML patients). The exosomal standard marker TSG101 was used as the internal control standard. The levels of RPL14, and RPL13 were significantly higher in IM-R CML patients compared with IM-S CML patients, which was consistent with proteomics and PRM results. The results are shown in **Figure 6** and **Table 1**.

**Table 1:** The results of PRM.

Protein Gene	CML_AP Normalized Area	CML_BP Normalized Area	CML_CP Normalized Area	CML_S2 Normalized Area	CML_S3 Normalized Area	R/S Ratio	R/SP value
RANBP1	0.36	1.39	Inf-	0.15	Inf-	5.73	Inf-
RPLP2	0.86	1.95	1.17	0.18	0.11	9.00	4.60E-03
ENO2	2.20	0.03	2.56	0.02	0.00	115.24	7.24E-02
USP5	2.84	0.32	0.05	0.05	0.23	7.73	2.58E-01
PSMA4	0.93	0.31	0.20	0.03	0.15	5.35	6.74E-02
CASP14	1.11	1.06	0.54	2.14	1.06	0.56	1.26E-01
NACA	0.57	1.43	Inf-	0.12	Inf-	8.60	Inf-
IKBKB	3.26	0.57	0.03	Inf-	Inf-	20.00	Inf-
RPL14	0.36	0.49	2.52	Inf-	Inf-	20.00	Inf-
RPL13	1.36	1.13	1.00	Inf-	Inf-	20.00	Inf-
RPS15A	0.05	0.12	0.07	Inf-	Inf-	20.00	Inf-
EIF3B	1.55	0.95	0.05	0.62	0.12	2.27	3.97E-01
RPL22	0.71	1.23	Inf-	0.17	Inf-	5.54	Inf-

CML, chronic myeloid leukemia; CP, chronic phase; BP, blastic phase; AP, acceleration phase; R, imatinib-resistant chronic myeloid leukemia patients; S, imatinib-sensitive chronic myeloid leukemia patients; Inf-, infinitesimal.

## Discussion

The development of IM resistance is a significant clinical challenge for the treatment of CML (26). Fully understanding the mechanisms of resistance to IM will help us to overcome this problem in the future. The point mutation or amplification of *BCR-ABL* gene are the main resistant mechanisms to IM (27). Meanwhile, growing studies have shown that exosomes were linked with drug resistance (28, 29), by which exosomes from chemoresistant cells can transfer resistance to chemosensitive cells in a variety of tumors (30, 31), including CML (13, 14). Furthermore, Exosomes contain various specific substances from target cells and are stable in various bodily fluids including blood, urine, saliva, ascites fluids, and cerebrospinal, making them ideal biomarkers for liquid biopsies. Multiple studies have shown that exosomal protein markers with diagnostic significance of chemoresistance have been obtained in different tumors such as Prostate Cancer (32, 33), Breast Cancer (34), and B Cell Lymphoma (35). One study demonstrated that exosomes released by MSCs transferred their proteasome inhibitors resistance phenotype to the multiple myeloma cells, through PSMA3 and PSMA3-AS1 present in the cargo of exosome. Plasma exosomal proteins PSMA3 and PSMA3-AS1 might be a promising therapeutic targets and prognostic predictors for proteasome inhibitors resistance (36). Moreover, hypoxia-induced exosomes contain PKM2 protein, which were able to modulate resistance to cisplatin. Exosomal PKM2 may serve as a promising predictive biomarker for cisplatin treatment response in NSCLC (37). However, the plasma exosomes proteome in drug resistance of CML have rarely been investigated. Here, we performed a comparative proteomic analysis of the plasma exosomes from IM-R and IM-S CML patient in order to identify proteins that may have a relevant role for IM resistance in CML.

In this study, we first isolated plasma exosomes and comprehensively characterized them using multiple methods. The TEM results shown that the morphological structure and size of plasma exosomes were consistent with the typical exosomes. Western blot revealed that the representative markers of exosomes, such as CD81, TSG101, and ALIX were detected in plasma exosomes. These results shown that we successfully isolated exosomes from plasma of IM-R and IM-S CML patients by ultracentrifugation. Furthermore, we established a solid foundation to facilitate further investigations of the exosome proteomic of CML. Then we carried out proteomic study on the plasma exosome by label free quantitation based on LC-MS/MS and identified IM-resistance associated potential biomarkers. The proteome analysis revealed 2654 quantifiable proteins in all groups. Out of the total 2654 proteins, 2005 (75.55%) proteins were found in the ExoCarta and Vesiclepedia databases, providing further evidence for the identity of isolated plasma exosomes. In addition, 234 (8.82%) proteins are not present in the ExoCarta and Vesiclepedia databases, which may be newly discovered exosomal proteins. Certainly, this assumption needs to be further validated. There were 279 DEPs with 151 upregulated and 128 downregulated proteins in IM-R CML patients compared with IM-S CML patients. The GO and KEGG functional analysis shown that the upregulated and downregulated proteins were related to ribosome and lipid metabolism, respectively. These DEPs were helpful in illustrating the resistance mechanisms of IM in CML.

To screen resistance biomarker to IM in CML, we further analyzed the DEPs. Sixteen hub proteins were screened from DEPs through the PPI network and the cytoHubba plugin of Cytoscape software. Among the 16 hub proteins, 4 proteins (RPL14, RPL18A, RPS15A, and RPL13) were expressed only in the drug-resistant group and selected as candidate biomarker for further studies. PRM verification analysis is a high throughput quantitative technique with high accuracy (38). Twenty proteins including RPL14, RPL18A, RPS15A, and RPL13 were selected for further verification by PRM quantification. 13 out of 20 proteins were detected and the expression trend was consistent with the results of MS, indicating the good reliability of the proteomic profiling. Finally, we selected two proteins for validation by WB in an independent cohort with 18 resistant and 18 sensitive CML. Surprisingly, the results confirmed that exosomal RPL13 and RPL14 levels in IM-R CML patients were significantly higher than IM-S CML patients.

Ribosomal proteins are central components of the ribosome and are involved in protein biosynthesis. However, extraribosomal functions of ribosomal proteins were gradually recognized (39, 40). Recent studies have found that ribosomal proteins were closely associated with tumorigenesis (41), tumor development (42), and resistance to antitumor drugs (43). For example, the expression of RPL34 was found to be more highly in osteosarcoma tissues compared with normal adjacent tissue and was correlated with a poor prognosis (44). RPL41 can induce the degradation of the activating transcription factor 4 (ATF4) to regulate tumour cell death, cell cycle, and chemosensitivity. Therefore, RPL41 may have potential as an anti-ATF4 agent for cancer therapy (45). These studies strongly support that the ribosomal protein might act as biomarkers and therapeutic targets for tumor.

RPL13 and RPL14 belong to ribosomal proteins and are a component of the large subunit of the ribosome. RPL13 was highly expressed in gastrointestinal cancer and enhanced the proliferation of gastrointestinal cancer cells and may be a useful molecular marker of drug efficacy in gastrointestinal cancer (46). In kidney renal clear cell carcinoma (KRCCC), researchers found that RPL13 was associated with the prognosis of tumor patients (47). Huang found that RPL14 are frequently altered during tumorigenesis of esophageal squamous cell carcinomas (ESCC) and could be used for the early detection of ESCC (48). In addition, RPL14 may be a tumor suppressor gene in nasopharyngeal carcinoma (NPC), which could inhibit cancer progression (49). Bioinformatics analyses suggests that the low expressions of ribosomal protein L14 (RPL14) indicated poor prognosis in Triple-negative breast cancer (TNBC) (50). In contrast to the above studies, some studies have shown that RPL14 may be an oncogenes, down-regulation of RPL14 could inhibit the development of cervical cancer (51), and high expression of RPL14 could promote the migration and invasion of cervical cancer cells (52). Thus, ribosomal proteins may play different functions in different tumors. In our study, we found that plasma exosome proteins RPL13 and RPL14 were highly expressed in IM-R CML patients, and could be used as potential markers for predicting and monitoring IM response in CML patients. However, whether it was involved in IM resistance in patients with CML and its corresponding mechanism remains to be further confirmed.

There are some unavoidable flaws in our study. For example, the exosomes in plasma are derive from various cells in the human body, including tumor cells and non-tumor cells. Therefore, the exosomes we extracted from patient plasma were non-tumor-specific, with strong heterogeneity from various cell. Simultaneously, due to the limitations of extraction methods of exosomes, we cannot exclude the possibility of the extracted exosomes being mixed with contaminated proteins or other particles. These heterogeneous exosomes or contaminants may have some impact on the experimental results, which are unavoidable problems (common problems) in the current field of exosome research. On the other hand, the cohort used to verify marker proteins was a relatively small number of patients from a single center, which is not enough to ensure the diagnostic efficacy of the screened marker proteins. Our results will need further verification with large samples from multi-centers in the future.

## Conclusion

In summary, we successfully isolated and characterized plasma exosomes from CML patients. Meanwhile, we also first identified and compared the global proteomic profiles of plasma exosomes between IM-R and IM-S patients. Four significant proteins were screened and two of them (RPL13 and RPL14) were validated as novel potential biomarkers of IM-resistance in CML. These findings will provide new ideas and important information for further studies of IM resistance in CML.

## Data Availability Statement

The datasets presented in this study can be found in online repositories. The names of the repository/repositories and accession number(s) can be found below: PRIDE under accession number is PXD028779.

## Ethics Statement

The studies involving human participants were reviewed and approved by the Medical Ethics Committee of the Second Affiliated Hospital of Nanchang University. The patients/participants provided their written informed consent to participate in this study.

## Author Contributions

BH and X-ZW designed the study. M-YL, CZ, LC, F-YY, Q-HM, JLin, H-BZ, and JLiu were responsible for the collection of the clinical samples. M-YL, CZ, YC, SX, and Y-LY performed the experiments. M-YL, F-MZ, and J-YJ analyzed the data. M-YL wrote the main manuscript. BH and X-ZW contributed to manuscript review and revision. BH and X-ZW contributed equally to this work. All authors contributed to the article and approved the submitted version.

## Funding

This study was supported by the National Natural Science Foundation of China (No. 81660029, No. 81860034), Jiangxi Provincial Innovation Special Fund Project for Postgraduates (No. YC2020-B036), and the Health Commission Technology Program of Jiangxi province (No. 202130348).

## Conflict of Interest

The authors declare that the research was conducted in the absence of any commercial or financial relationships that could be construed as a potential conflict of interest.

## Publisher’s Note

All claims expressed in this article are solely those of the authors and do not necessarily represent those of their affiliated organizations, or those of the publisher, the editors and the reviewers. Any product that may be evaluated in this article, or claim that may be made by its manufacturer, is not guaranteed or endorsed by the publisher.

## Abbreviations

IM, imatinib; TKI, tyrosine kinase inhibitor; CML, chronic myeloid leukemia; LC-MS/MS, liquid chromatograph-mass spectrometer; WB, western blot; PRM, parallel reaction monitoring; DEPs, differentially expressed proteins; Ph, Philadelphia; EVs, extracellular vesicles; MVBs, multivesicular bodies; IM-R, imatinib-resistant; IM-S, imatinib-sensitive; UC, ultracentrifuge; NTA, nanoparticle tracking analysis; TEM, Transmission Electron Microscope; SDS-PAGE, sodium dodecyl sulfate-polyacrylamide gel electrophoresis; GO, Gene Ontology; KEGG, Genes and Genomes.

## References

[1] Nash I. Chronic Myeloid Leukemia. N Engl J Med (1999) 341:765. doi: 10.1056/NEJM199909023411016 10475801

[2] Goldman JM Melo JV. Targeting the BCR-ABL Tyrosine Kinase in Chronic Myeloid Leukemia. N Engl J Med (2001) 344:1084–6. doi: 10.1056/NEJM200104053441409 11287980

[3] Oehler VG Gooley T Snyder DS Johnston L Lin A Cummings CC. The Effects of Imatinib Mesylate Treatment Before Allogeneic Transplantation for Chronic Myeloid Leukemia. Blood (2007) 109:1782–89. doi: 10.1182/blood-2006-06031682 PMC1794075 17062727

[4] Hochhaus A Larson RA Guilhot F Radich JP Branford S Hughes TP. Long-Term Outcomes of Imatinib Treatment for Chronic Myeloid Leukemia. N Engl J Med (2017) 376:917–27. doi: 10.1056/NEJMoa1609324 PMC5901965 28273028

[5] Kalluri R LeBleu VS. The Biology, Function, and Biomedical Applications of Exosomes. Sci (2020) 367:eaau6977. doi: 10.1126/science.aau6977 PMC7717626 32029601

[6] van Niel G D'Angelo G Raposo G. Shedding Light on the Cell Biology of Extracellular Vesicles. Nat Rev Mol Cell Biol (2018) 19:213–28. doi: 10.1038/nrm.2017.125 29339798

[7] Johnstone RM Mathew A Mason AB Teng K. Exosome Formation During Maturation of Mammalian and Avian Reticulocytes_ Evidence That Exosome Release is a Major Route for Externalization of Obsolete Membrane Proteins. J Cell Physiol (1991) 147:27–36. doi: 10.1002/jcp.1041470105 2037622

[8] Dou G Tian R Liu X Yuan P Ye Q Liu J. Chimeric Apoptotic Bodies Functionalized With Natural Membrane and Modular Delivery System for Inflammation Modulation. Sci Adv (2020) 6:eaba2987–eaba87. doi: 10.1126/sciadv.aba2987 PMC7439513 32832662

[9] Tkach M Thery C. Communication by Extracellular Vesicles: Where We Are and Where We Need to Go. Cell (2016) 164:1226–32. doi: 10.1016/j.cell.2016.01.043 26967288

[10] Gowda R Robertson BM Iyer S Barry J Dinavahi SS Robertson GP. The Role of Exosomes in Metastasis and Progression of Melanoma. Cancer Treat Rev (2020) 85:101975. doi: 10.1016/j.ctrv.2020.101975 32050108

[11] Maacha S Bhat AA Jimenez L Raza A Haris M Uddin S. Extracellular Vesicles-Mediated Intercellular Communication: Roles in the Tumor Microenvironment and Anti-Cancer Drug Resistance. Mol Cancer (2019) 18:55. doi: 10.1186/s12943-019-0965-7 30925923 PMC6441157

[12] Mashouri L Yousefi H Aref AR Ahadi AM Molaei F Alahari SK. Exosomes: Composition, Biogenesis, and Mechanisms in Cancer Metastasis and Drug Resistance. Mol Cancer (2019) 18:75. doi: 10.1186/s12943-019-0991-5 30940145 PMC6444571

[13] Min QH Wang XZ Zhang J Chen QG Li SQ Liu XQ. Exosomes Derived From Imatinib-Resistant Chronic Myeloid Leukemia Cells Mediate a Horizontal Transfer of Drug-Resistant Trait by Delivering miR-365. Exp Cell Res (2018) 362:386–93. doi: 10.1016/j.yexcr.2017.12.001 29223442

[14] Hrdinova T Toman O Dresler J Klimentova J Salovska B Pajer P. Exosomes Released by Imatinibresistant K562 Cells Contain Specific Membrane Markers, IFITM3, CD146 and CD36 and Increase the Survival of Imatinibsensitive Cells in the Presence of Imatinib. Int J Oncol (2021) 58:238–50. doi: 10.3892/ijo.2020.5163 33491750

[15] Arber DA Orazi A Hasserjian R Thiele J Borowitz MJ Le Beau MM. The 2016 Revision to the World Health Organization Classification of Myeloid Neoplasms and Acute Leukemia. Blood (2016) 127:2391–405. doi: 10.1182/blood-2016-03-643544 27069254

[16] Chinese Society of Hematology CMA. The Guidelines for Diagnosis and Treatment of Chronic Myelogenous Leukemia in China (2016 Edition). Zhonghua Xue Ye Xue Za Zhi (2016) 37:633–9. doi: 10.3760/cma.j.issn.0253-2727.2016.08.001 PMC7348542 27587241

[17] Jiang YH Liu J Lin J Li SQ Xu YM Min QH. K562 Cell-Derived Exosomes Suppress the Adhesive Function of Bone Marrow Mesenchymal Stem Cells *via* Delivery of miR-711. Biochem Biophys Res Commun (2020) 521:584–89. doi: 10.1016/j.bbrc.2019.10.096 31677790

[18] Hou G Harley ITW Lu X Zhou T Xu N Yao C. SLE non-Coding Genetic Risk Variant Determines the Epigenetic Dysfunction of an Immune Cell Specific Enhancer That Controls Disease-Critical microRNA Expression. Nat Commun (2021) 12:135. doi: 10.1038/s41467-020-20460-1 33420081 PMC7794586

[19] Yu G Wang LG Han Y He QY. Clusterprofiler: An R Package for Comparing Biological Themes Among Gene Clusters. OMICS (2012) 16:284–7. doi: 10.1089/omi.2011.0118 PMC3339379 22455463

[20] Szklarczyk D Gable AL Lyon D Junge A Wyder S Huerta-Cepas J. STRING V11: Protein-Protein Association Networks With Increased Coverage, Supporting Functional Discovery in Genome-Wide Experimental Datasets. Nucleic Acids Res (2019) 47:D607–13. doi: 10.1093/nar/gky1131 PMC6323986 30476243

[21] Otasek D Morris JH Boucas J Pico AR Demchak B. Cytoscape Automation: Empowering Workflow-Based Network Analysis. Genome Biol (2019) 20:185. doi: 10.1186/s13059-019-1758-4 31477170 PMC6717989

[22] Mathivanan S Fahner CJ Reid GE Simpson RJ. ExoCarta 2012: Database of Exosomal Proteins, RNA and Lipids. Nucleic Acids Res (2012) 40:D1241–4. doi: 10.1093/nar/gkr828 PMC3245025 21989406

[23] Keerthikumar S Chisanga D Ariyaratne D Al Saffar H Anand S Zhao K. ExoCarta: A Web-Based Compendium of Exosomal Cargo. J Mol Biol (2016) 428:688–92. doi: 10.1016/j.jmb.2015.09.019 PMC4783248 26434508

[24] Kalra H Simpson RJ Ji H Aikawa E Altevogt P Askenase P. Vesiclepedia: A Compendium for Extracellular Vesicles With Continuous Community Annotation. PloS Biol (2012) 10:e1001450. doi: 10.1371/journal.pbio.1001450 23271954 PMC3525526

[25] Pathan M Fonseka P Chitti SV Kang T Sanwlani R Van Deun J. Vesiclepedia 2019: A Compendium of RNA, Proteins, Lipids and Metabolites in Extracellular Vesicles. Nucleic Acids Res (2019) 47:D516–19. doi: 10.1093/nar/gky1029 PMC6323905 30395310

[26] Lei H Xu HZ Shan HZ Liu M Lu Y Fang ZX. Targeting USP47 Overcomes Tyrosine Kinase Inhibitor Resistance and Eradicates Leukemia Stem/Progenitor Cells in Chronic Myelogenous Leukemia. Nat Commun (2021) 12:51. doi: 10.1038/s41467-020-20259-0 33397955 PMC7782553

[27] Gorre ME Mohammed M Ellwood K Hsu N Paquette R Nagesh Rao P. Clinical Resistance to STI-571 Cancer Therapy Caused by BCR-ABL Gene Mutation or Amplification. Sci (American Assoc Advancement Sci) (2001) 293:876–80. doi: 10.1126/science.1062538 11423618

[28] Namee NM O'Driscoll L. Extracellular Vesicles and Anti-Cancer Drug Resistance. Biochim Biophys Acta Rev Cancer (2018) 1870:123–36. doi: 10.1016/j.bbcan.2018.07.003 30003999

[29] Milman N Ginini L Gil Z. Exosomes and Their Role in Tumorigenesis and Anticancer Drug Resistance. Drug Resist Updat (2019) 45:1–12. doi: 10.1016/j.drup.2019.07.003 31369918

[30] Wang X Cheng K Zhang G Jia Z Yu Y Guo J. Enrichment of CD44 in Exosomes From Breast Cancer Cells Treated With Doxorubicin Promotes Chemoresistance. Front Oncol (2020) 10:960. doi: 10.3389/fonc.2020.00960 32760666 PMC7373100

[31] Ding C Yi X Wu X Bu X Wang D Wu Z. Exosome-Mediated Transfer of circRNA CircNFIX Enhances Temozolomide Resistance in Glioma. Cancer Lett (2020) 479:1–12. doi: 10.1016/j.canlet.2020.03.002 32194140

[32] Kawakami K Fujita Y Kato T Mizutani K Kameyama K Tsumoto H. Integrin Beta4 and Vinculin Contained in Exosomes are Potential Markers for Progression of Prostate Cancer Associated With Taxane-Resistance. Int J Oncol (2015) 47:384–90. doi: 10.3892/ijo.2015.3011 25997717

[33] Kato T Mizutani K Kameyama K Kawakami K Fujita Y Nakane K. Serum Exosomal P-Glycoprotein is a Potential Marker to Diagnose Docetaxel Resistance and Select a Taxoid for Patients With Prostate Cancer. Urol Oncol (2015) 33:385 e15–20. doi: 10.1016/j.urolonc.2015.04.019 26027763

[34] Yang SJ Wang DD Li J Xu HZ Shen HY Chen X. Predictive Role of GSTP1-Containing Exosomes in Chemotherapy-Resistant Breast Cancer. Gene (2017) 623:5–14. doi: 10.1016/j.gene.2017.04.031 28438694

[35] Feng Y Zhong M Tang Y Liu X Liu Y Wang L. The Role and Underlying Mechanism of Exosomal CA1 in Chemotherapy Resistance in Diffuse Large B Cell Lymphoma. Mol Ther Nucleic Acids (2020) 21:452–63. doi: 10.1016/j.omtn.2020.06.016 PMC7358223 32668392

[36] Xu H Han H Song S Yi N Qian C Qiu Y. Exosome-Transmitted PSMA3 and PSMA3-AS1 Promote Proteasome Inhibitor Resistance in Multiple Myeloma. Clin Cancer Res (2019) 25:1923–35. doi: 10.1158/1078-0432.CCR-18-2363 30610101

[37] Wang D Zhao C Xu F Zhang A Jin M Zhang K. Cisplatin-Resistant NSCLC Cells Induced by Hypoxia Transmit Resistance to Sensitive Cells Through Exosomal PKM2. Theranostics (2021) 11:2860–75. doi: 10.7150/thno.51797 PMC7806469 33456577

[38] Bourmaud A Gallien S Domon B. Parallel Reaction Monitoring Using Quadrupole-Orbitrap Mass Spectrometer: Principle and Applications. Proteomics (2016) 16:2146–59. doi: 10.1002/pmic.201500543 27145088

[39] Wool IG. Extraribosomal Functions of Ribosomal Proteins. Trends Biochem Sci (1996) 21:164–5. doi: 10.1016/S0968-0004(96)20011-8 8871397

[40] Warner JR McIntosh KB. How Common are Extraribosomal Functions of Ribosomal Proteins? Mol Cell (2009) 34:3–11. doi: 10.1016/j.molcel.2009.03.006 19362532 PMC2679180

[41] Liu PY Tee AE Milazzo G Hannan KM Maag J Mondal S. The Long Noncoding RNA Lncnb1 Promotes Tumorigenesis by Interacting With Ribosomal Protein RPL35. Nat Commun (2019) 10:5026. doi: 10.1038/s41467-019-12971-3 31690716 PMC6831662

[42] Ebright RY Lee S Wittner BS Niederhoffer KL Nicholson BT Bardia A. Deregulation of Ribosomal Protein Expression and Translation Promotes Breast Cancer Metastasis. Science (2020) 367:1468–73. doi: 10.1126/science.aay0939 PMC7307008 32029688

[43] Floristan A Morales L Hanniford D Martinez C Castellano-Sanz E Dolgalev I. Functional Analysis of RPS27 Mutations and Expression in Melanoma. Pigment Cell Melanoma Res (2020) 33:466–79. doi: 10.1111/pcmr.12841 PMC7180098 31663663

[44] Luo S Zhao J Fowdur M Wang K Jiang T He M. Highly Expressed Ribosomal Protein L34 Indicates Poor Prognosis in Osteosarcoma and Its Knockdown Suppresses Osteosarcoma Proliferation Probably Through Translational Control. Sci Rep (2016) 6:37690. doi: 10.1038/srep37690 27883047 PMC5121591

[45] Wang A Xu S Zhang X He J Yan D Yang Z. Ribosomal Protein RPL41 Induces Rapid Degradation of ATF4, a Transcription Factor Critical for Tumour Cell Survival in Stress. J Pathol (2011) 225:285–92. doi: 10.1002/path.2918 21706477

[46] Kobayashi T Sasaki Y Oshima Y Yamamoto H Mita H Suzuki H. Activation of the Ribosomal Protein L13 Gene in Human Gastrointestinal Cancer. Int J Mol Med (2006) 18:161–70. doi: 10.3892/ijmm.18.1.161 16786168

[47] Zhong W Huang C Lin J Zhu M Zhong H Chiang MH. Development and Validation of Nine-RNA Binding Protein Signature Predicting Overall Survival for Kidney Renal Clear Cell Carcinoma. Front Genet (2020) 11:568192. doi: 10.3389/fgene.2020.568192 33133154 PMC7566920

[48] Huang XP Zhao CX Li QJ Cai Y Liu FX Hu H. Alteration of RPL14 in Squamous Cell Carcinomas and Preneoplastic Lesions of the Esophagus. Gene (2006) 366:161–8. doi: 10.1016/j.gene.2005.09.025 16316724

[49] Zhang Z Zhang Y Qiu Y Mo W Yang Z. Human/eukaryotic Ribosomal Protein L14 (RPL14/eL14) Overexpression Represses Proliferation, Migration, Invasion and EMT Process in Nasopharyngeal Carcinoma. Bioengineered (2021) 12:2175–86. doi: 10.1080/21655979.2021.1932225 PMC8806664 34057029

[50] Lin Z Peng R Sun Y Zhang L Zhang Z. Identification of Ribosomal Protein Family in Triple-Negative Breast Cancer by Bioinformatics Analysis. Biosci Rep (2021) 41:BSR20200869. doi: 10.1042/BSR20200869 33305312 PMC7789804

[51] Sun N Zhang W Liu J Yang X Chu Q. Propofol Inhibits the Progression of Cervical Cancer by Regulating HOTAIR/miR-129-5p/RPL14 Axis. Onco Targets Ther (2021) 14:551–64. doi: 10.2147/OTT.S279942 PMC7829600 33505161

[52] Feng Y Ma J Fan H Liu M Zhu Y Li Y. TNF-Alpha-Induced lncRNA LOC105374902 Promotes the Malignant Behavior of Cervical Cancer Cells by Acting as a Sponge of miR-1285-3p. Biochem Biophys Res Commun (2019) 513:56–63. doi: 10.1016/j.bbrc.2019.03.079 30935691

